# Towards better metrics and policymaking for seed system development: Insights from Asia's seed industry

**DOI:** 10.1016/j.agsy.2016.05.015

**Published:** 2016-09

**Authors:** David J. Spielman, Adam Kennedy

**Affiliations:** International Food Policy Research Institute, 2033 K St NW, Washington, DC 20006, USA

**Keywords:** Seed systems, Seed markets, Input subsidies, Biosafety, Intellectual property rights, Asia

## Abstract

Since the 1980s, many developing countries have introduced policies to promote seed industry growth and improve the delivery of modern science to farmers, often with a long-term goal of increasing agricultural productivity in smallholder farming systems. Public, private, and civil society actors involved in shaping policy designs have, in turn, developed competing narratives around how best to build an innovative and sustainable seed system, each with varying goals, values, and levels of influence. Efforts to strike a balance between these narratives have often played out in passionate discourses surrounding seed rules and regulations. As a result, however, policymakers in many countries have expressed impatience with the slow progress on enhancing the contribution of a modern seed industry to the overarching goal of increasing agricultural productivity growth. One reason for this slow progress may be that policymakers are insufficiently cognizant of the trade-offs associated with rules and regulations required to effectively govern a modern seed industry. This suggests the need for new data and analysis to improve the understanding of how seed systems function. This paper explores these issues in the context of Asia's rapidly growing seed industry, with illustrations from seed markets for maize and several other crops, to highlight current gaps in the metrics used to analyze performance, competition, and innovation. The paper provides a finite set of indicators to inform policymaking on seed system design and monitoring, and explores how these indicators can be used to inform current policy debates in the region.

## Highlights

• Despite several decades of policy reforms, decisionmaking on seed systems in many developing countries remain poorly informed by market- and policy-relevant indicators.
• We explores Asia’s rapidly growing seed industry to illustrate current gaps in metrics used to analyze performance, competition, and innovation.
• We present a finite set of seed system indicators to inform policymaking.
• We discuss how these indicators might be used to shape competing narratives and inform current policy discourse around seed system develop in the region.

## 1 Introduction

Policymakers often face difficult challenges in promoting seed industry growth in developing countries where the intended beneficiaries are small-scale, resource-poor farmers operating in highly fragmented markets. Yet as a pathway to enhancing agricultural productivity and improving food security, there is strong historical evidence indicating that improved cultivars—and the seed systems required to deliver those cultivars to smallholders—are a highly effective means of doing so (Evenson and Gollin, 2003, Alston et al., 2000).

Despite the introduction of seed policy reforms beginning in the late 1980s, many developing-country policymakers still express concern or impatience with the slow progress on enhancing the contribution of a modern seed industry to the overarching goal of increasing agricultural productivity growth. One factor that has contributed to this situation is the enormous complexity in designing and implementing policies, rules and regulations that are appropriate to a given country's context, stage of development, needs, and priorities. Many of these early policy reforms during the 1980s tended to fall short because they equated market liberalization with seed system deregulation and privatization, leading to protracted struggles over the appropriate roles for the public and private sectors in cultivar improvement, and seed production and distribution to farmers (see, e.g., Tripp and Louwaars, 1997, Tripp, 1997, Byerlee and Echeverrıa, 2002).

One explanation for the persistence of this struggle may be that policies have been formulated and executed with insufficient cognizance of the trade-offs associated with rules and regulations designed to govern a modern seed industry. Where the aim is to supply affordable quantities of high-quality seed of improved cultivars to populations and markets made up of heterogeneous farmers and farming systems, there is no single set of rules or regulations that leads directly to the development of a system that is both productive and innovative across breeding, seed production, regulation, distribution, and marketing. Rather, decisions on how to build that system must balance a complex set of societal and economic trade-offs.

Static trade-offs exist, for example, in the distribution of the gains from innovation among plant breeders, entrepreneurs, seed companies, public research organizations, and farmers themselves (Kloppenburg, 1988, Jaffee and Srivastava, 1994, Morris et al., 1998). Intertemporal trade-offs exist where present efforts to introduce yield-enhancing cultivars threaten the in situ conservation of genetic diversity required to support future investments in cultivar improvement (Smale, 2006). Actors involved in these decisions necessarily develop competing narratives around how best to build an innovative and sustainable seed system, each with varying goals, values, and levels of influence. As a country's seed industry evolves and grows in size and value, balancing these narratives become increasingly difficult—but no less important (Tripp and Louwaars, 1997, Scoones and Thompson, 2011, Coomes et al., 2015).

In many developing countries, efforts to strike a balance between these narratives often plays out in the policy discourse surrounding national seed policies, rules and regulations. Often, however, the public policy discourse tends to overlook changing realities in the region's agricultural sector and seed systems. These changes include, inter alia, the rapid growth in private investment in cultivar improvement, biotechnology, and seed production and marketing (Pray and Fuglie, 2001, Langyintuo et al., 2010); stagnation in the capacity and contribution of public research to cultivar improvement (Beintema et al., 2012, Flaherty et al., 2013); and insufficient investment in the conservation of in situ and ex situ plant genetic resources (Koo et al., 2004, Smale, 2006). Instead, the policy discourse is often mired in legal and regulatory dimensions of seed systems management, with disproportional emphasis placed on the minutiae of rules, guidelines, procedures, protocols, and organizational mandates without commensurate analysis of their costs and benefits (see Tripp et al., 1997, Tripp and Louwaars, 1997). In some instances, the narrative revolves around the public regulator's emphasis on protecting the farmer from unfair business practices by seed providers, possibly resulting in a constrictive regulatory system. In other instances, the narrative may hinge on the social planner's desire for a more liberal economic system, which may come only with the withdrawal of regulations designed to protect farmers. In still others, the narrative may revolve around optimal ways of conserving scarce natural resources and biodiversity, resulting in an entirely different set of regulatory priorities and mechanisms.

One way of shifting this discourse is to focus attention on the data and analysis that expand the understanding of how seed systems function. This paper explores these issues in the context of Asia's rapidly growing seed sector. Specifically, the paper explores current gaps in the metrics used to analyze performance, competition, and innovation in the seed industry, with a specific emphasis on maize in selected Asian developing countries. It then describes and characterizes a finite set of indicators to inform policymaking on seed system design, and explores how these indicators can be used to inform current policy debates in the region.

This paper proceeds as follows. Section 2 provides background on the performance and growth of the maize seed markets in selected Asian countries to illustrate the relationship between innovation and competition in developing-country seed systems, with several caveats on the paper's wider applicability. Section 3 critiques conventional indicators used to measure seed industry performance before proposing alternative indicators and examining the feasibility of collecting data on these indicators. Section 4 illustrates the utility of the proposed indicators for current policy debates surrounding Asia's seed systems. Section 5 provides policy recommendations and concluding remarks.

## 2 Innovation, competition, and maize

The present analysis relies partly on an industrial organization perspective on seed system development—a perspective that is slowly gaining currency in the study of agricultural development (Reardon and Timmer, 2012)—to illustrate the importance of measuring relationships between performance, innovation, and competition. For several reasons, maize provides an opportunity to demonstrate the utility of this perspective and the applicability of indicators that measure and monitor seed system development.

First, and unlike most other major field crops, maize has historically attracted significant levels of private investment in research, production, and marketing. Maize's appeal stems primarily from the breeder's ability to induce the expression of heterosis—an increase in yield or uniformity that results from genetic contributions derived from the crossing of distinct parental lines—in maize hybrids. This translates into economic value for breeders and seed companies because the yield gains conferred by heterosis decline dramatically after the first generation of hybrid seed (F1) is planted, thus compelling farmers to purchase new F1 seed each season to continually realize these gains. This is in contrast to the much lower economic value created by improving open-pollinated maize varieties (or by improving self-pollinated crops such as rice and wheat), from which harvested grain can be saved for use as seed in the subsequent season. In essence, the reproductive biology of hybrid maize confers a biological form of intellectual property rights protection to the breeder, creating an innovation incentive that has been central to fuelling a century of global knowledge accumulation in maize improvement in both industrialized and developing countries (Byerlee and Eicher, 1997, Morris, 1998, Fernandez-Cornejo, 2004). Estimates place the global market for maize seeds and traits at approximately $5 billion in 2006 and $10–12 billion in 2012, with the associated spending on R&D ranging from $1 to $4 billion (Fuglie et al., 2011, Bonny, 2014).^1^ These figures are far greater than those for any other food staple crop.

Second, maize is an appealing crop to focus on given the rapid growth in demand for its use as feed for the livestock and poultry industries that supply Asia's rising population of consumers with incomes to spend on higher-value foods (Gulati and Dixon, 2008). The rapid growth in the derived demand for maize requires well-functioning markets for both maize seed and grain, a robust innovation system around maize improvement, and an effective regulatory system to sustain an innovative and competitive market. Yet in many developing countries, not all of these elements are in place, particularly the enabling policy environment needed to promote sustainable intensification of maize production among smallholders in Asia (Gerpacio and Pingali, 2007).

Third, earlier work on seed markets in Asia points to the maize seed industry's rapid growth as a “success story”^2^ in which policy reforms introduced from the 1980s onwards succeeded in opening the market to private seed companies (Morris, 1998, Pray and Fuglie, 2001). Multinational seed companies with strong R&D programs and product lines played a central role in these markets, operating independently or in joint ventures with domestic seed companies in India (e.g., Joshi et al., 2005; Pray and Nagarajan, 2014), Pakistan (Rana, 2014), Thailand (Napasintuwong, 2014), and elsewhere. In India, for example, liberalization of seed market policy during the late 1980s encouraged the rapid growth of a private sector-led maize seed industry which, in turn, fuelled significant yield growth in maize (Morris et al., 1998, Pal et al., 1998; Pray et al., 2001; Ramaswami, 2002). The effects of this industry growth have been so substantial that the annual growth rates of yield, output, and area under maize cultivation during the period 2004–05 to 2013–14 were 2.9, 2.5, and 5.5%, respectively (KPMG/FICCI/NCDEX, 2014). Thailand experienced a similar growth pattern in which the combination of policy reforms and a strong public-sector maize development program in the 1970s transitioned the country into a hub for private R&D investment (see Fuglie, 2001, Napasintuwong, 2014).

Fourth, this rapid growth in industry size and innovation raises policy-relevant questions about market competition and concentration. Market concentration has been increasing in the global markets for seeds and traits (Fuglie et al., 2011), with some evidence of similar trends in countries such as India (Spielman et al., 2014). Yet there is little immediate evidence from these studies that concentration has led to significant changes in private R&D spending or R&D intensities which might, in turn, affect the rate of innovation in maize improvement. Rather, these studies imply that there is still room for growth in both R&D spending and industry size, and that issues of monopolistic pricing, cartels, and other signs of a non-competitive market are second-order concerns at present. Still, these issues remain central to the policy discourse, implying that they are still an important area of analysis for the region.

Finally, the rapid growth of private sector leadership in Asia's maize seed market has not entirely replaced the need for public research. Through the 1990s, national maize breeding programs remained an active source of locally adapted materials, particularly improved open-pollinated varieties (Gerpacio, 2003).^3^ And even today, many Asian countries retain maize breeding programs and maize seed production and distribution programs, often with a mandate to supply underserved populations with open-pollinated varieties. However, given that public programs on maize R&D must compete for scarce funds with programs for other crops and other research priorities, and given that many public research systems are struggling with scientific capacity limitations, volatile funding trends, top-heavy organizational structures, and weak research incentives, questions remain about the proper role for the public sector in maize research.^4^

That said, maize is a means to an end in the present analysis—a vehicle to address wider issues of seed market development in Asia. This implies that there are limits on the applicability of the discussion on indicators and metrics that follows. In particular, these indicators tend to be immediately relevant only to a *commercial* seed industry. This implies that the present analysis is primarily relevant to crops where innovators can appropriate the gains from innovation, whether through reproductive biology, intellectual property rights, or some combination of technological and institutional mechanisms. Such is typically not the case for wheat, rice, many grain legumes, and vegetatively propagated root and tuber crops. Seed markets for these crops rely significantly on largely state-led production and distribution systems or non-industrial systems in which farmers save, select, and exchange seeds among themselves (e.g., Lipper et al., 2010). Many of these crops are also cultivated within regions of genetic origin or diversity, highlighting the importance of informal seed systems as a means of both cultivar improvement and genetic conservation that are somewhat less applicable to maize, an exotic crop with limited genetic diversity in Asia (De Boef et al., 2010, Coomes et al., 2015).^5^

However, this analysis does not overlook the role of farmers—especially smallholder farmers—in a modern seed system. The overlap and integration between formal and informal seed markets in many developing countries means that farmers play multiple roles in a modern seed system. They function as outgrowers for formal seed producers; conduits for new cultivar dissemination from the formal to informal system; innovators through traditional variety selection and seed saving and through more formalized participatory breeding, varietal selection, and action research programs; and as in situ conservators of genetic material for future breeding programs (Almekinders and Louwaars, 2002, Sperling and McGuire, 2010, Louwaars and de Boef, 2012, Coomes et al., 2015).

This analysis is also relevant to a range of other crops for which hybrids have been successfully developed and commercialized, or for which investments in hybridization are ongoing. Well-documented examples from developing countries include sorghum, pearl millet, and cotton in India (Pray and Nagarajan, 2010; Gruère and Sun, 2012), rice in China (Li et al., 2010), hybrid rice in India and Bangladesh (Spielman et al., 2014), and a range of horticultural crops. For these reasons, the analysis occasionally ventures into the analysis of crops and technologies beyond maize hybrids.

Finally, it is important to point out that while the analysis examines indicators that are potentially useful in evaluating seed industry development, it is not a naïve treatise on the primacy of evidence in shaping policy change processes. Necessarily, evidence is not the only means by which policies change: the relationships between evidence and policy outcomes often hinge on highly context-specific political economy factors that affect policymaking processes (Mayer et al., 2012, Carden, 2004, Court and Maxwell, 2005, Kristjanson et al., 2009). As such, the conceptual framework, indicators, and analysis presented here are meant to stimulate discussion around investment in and regulation of seed systems, rather than provide a definitive statement on measuring and governing seed industry development. Much of this discussion has been put forth by others, both in general terms (e.g., Tripp et al., 1997, Morris, 1998, Almekinders and Louwaars, 2002, Morris and Heisey, 2003), and with respect to maize in Asia (Gerpacio, 2003, Pray and Ramaswami, 2001, Gerpacio and Pingali, 2007). However the literature would still benefit from a more concise assessment of the indicators and metrics that can inform policy change. The analysis that follows attempts to address this need.

## 3 Seed industry indicators and applications to selected Asian countries

In this section, we examine current practice in assessing seed industry performance to highlight the shortcomings of key indicators for ongoing policy discourse around seed industry development. We then examine a set of alternative indicators to measure competition and innovation and address the feasibility of obtaining data for these indicators.

### 3.1 Current practice

Conventional indicators used by policymakers to assess seed industry performance in many developing countries tend to be limited in terms of analytical value. Such indicators include quantity of seed produced, which is typically crop-specific data assembled from sector- or industry-level reports; estimates of the quantity of seed demanded, typically derived from crop-specific estimates of area under cultivation multiplied by recommended seeding rates; and shortfalls and surpluses between estimated supply and demand, calculated from a comparisons of these two indicators (Table 1). These highly aggregated indicators are rarely useful as they tend to be based on broad assumptions, empirical data that are not regularly updated, or some combination thereof. They lack insight into variety-level demand and supply quantities, as well as variation around mean quantities that may be induced by market and weather risk—all of which are critical to forecasting future supply and demand and planning research, production, and marketing (see, e.g., Burer et al., 2008).

Other conventional indicators are equally limited in analytical value. For example, seed replacement rates are commonly used to measure the proportion of seed that farmers purchase from the formal market rather than from their own saved or locally exchanged seed. They are typically based on aggregated national and sub-national data typically assembled from sector- or industry-level reporting, and contain and implicit assumption that purchased (i.e., certified or truthfully labeled seed) is inherently superior to farmer saved seed because farmers are thought to rely on poor selection, storage and preservation practices that lead to lower purity and germination rates or losses in genetic integrity when the seeds are used in cultivation.

In fact, results from several studies call this assumption into question. Bishaw et al. (2012) found that the physical purity and germination rates of recycled wheat and barley seed in Ethiopia and Syria were not significantly different from that of certified seed. Similarly, Biemond et al. (2013) found that Nigerian farmers' recycled seed was of poor quality but not more so than the seed being produced by public institutes such that neither passed the National Agriculture Seed Council's standards for certified seed. Deu et al. (2014) have found that with proper training in seed production, farmers are generally able to maintain the phenotype of their varieties and minimize off-type plants.

At best, the seed replacement rate indicates whether farmers are realizing the benefits conferred by F1 hybrids, suggesting that it does have some relevance when applied to crops that are not self-pollinating or vegetatively propagated. But a singular focus on this measure—as is common practice in many developing countries—tends to obscure the critical difference between seed replacement (improving the quality of inputs by purchasing fresh seed of either the same variety/hybrid or a new one) and varietal replacement (changing the genetic quality of an input by replacing seed of an older variety/hybrid with seed of a new one) (e.g., Brennan and Byerlee, 1991). The former provides a basic sense of industry sales volumes and market size, while latter provides a more meaningful measure of a seed industry's performance in terms of supplying improved products to farmers.

Yet even when taken together, these conventional indicators are still of limited analytical value if the distributional consequences of technological change are of concern to policymakers. Aggregate replacement and turnover rates are incomplete without additional data on who actually purchases seed—what type of farmers in terms of land tenure, wealth, income, or geographic location—and how alternative uses of public resources might change those distributional outcomes in a welfare-improving manner. In short, policy and investment decisions taken to maximize specific “rates” may be misinformed, potentially allocating scarce public resources to ambiguous ends.

Instead of relying on conventional indicators with limited analytical value, we suggest a finite set of alternative indicators designed to help policymakers and other actors understand seed industry structure and performance and pursue policies and investments in support of industry growth. Our suggested indicators for seed industry performance aim to answer the question of whether the institutional architecture of a seed system is efficient, effective, and dynamic enough to deliver benefits to farmers. To answer this question, better information is needed on seed industry structure, innovation, regulation, and performance along the lines of indicators used to assess the state of agricultural input industries in industrialized countries (see, e.g., Fernandez-Cornejo (2004) on the United States' seed industry), but adapted to fit the precise needs of countries with significantly different agricultural systems. These indicators are summarized in Table 2 and discussed in detail below.

### 3.2 Seed industry performance

Ideally, a basic set of performance indicators should provide insight not only into quantity—the volumes and values of seed supplied and demanded—but also the accessibility and quality of the seed. These indicators might include variety- and source-specific quantities of seed sold, the prices at which seeds are sold, area planted to improved varieties and changes in area planted over time (Morris and Heisey, 2003). They might include detailed geo-referencing to allow for spatial analysis of market coverage and participation. And they might include additional detail on farm size and social and economic characteristics of farmers who purchase the seed to better analyze market coverage, estimate of the benefits associated with adoption, and explore heterogeneity and distributional issues associated with adoption.

Necessarily, discussion of these indicators needs to account for the feasibility of collecting the data required to generate these indicators. Consider several initiatives that aim to accomplish precisely this outcome which attempt to improve on or systematize the conventional but ad hoc use of data collection through household, market/industry, or expert opinion surveys (Morris and Heisey, 2003). The first methodical attempt to measure seed system performance is highlighted by two projects led by the Consultative Group on International Agricultural Research (CGIAR) and its national research partners to assemble and analyze variety-specific cultivar diffusion data for 20 crops in more than 30 countries in Africa south of the Sahara and South Asia (see DIIVA (Diffusion and Impact of Improved Varieties in Africa, TRIVSA (Tracking Improved Varieties in South Asia project)). These projects provide a novel collection of data on scientific strength in national breeding programs, varietal releases, and adoption (measured as estimates in the share of area under each variety). Data are garnered from a combination of expert interviews and survey data, and provide the first searchable online database containing variety-specific information over both space and time. Unfortunately, these projects focus—by design—on public research achievements and provide little descriptive insight into the contributions of private industry, or the pathways through which public R&D is handed off to private seed companies, farmers' organizations, and other seed system actors.

Another recent attempt that allows for measurement of seed system performance, albeit indirectly, is the Living Standards Measurement Study-Integrated Surveys on Agriculture (LSMS-ISA), a project of the World Bank and national statistical agencies in seven Sub-Saharan countries (World Bank, 2015). The project's primary aim is to improve household and community data collection on agriculture, and the LSMS-ISA modules on seed use includes a range of questions that can potentially improve the resolution of data on what farmers sow, how much they use, what price they pay, and how they source their seed at the farm-, plot-, and variety-specific levels. LSMS-ISA data are geo-referenced and designed to generate panel datasets, allowing for potentially useful analysis over both time and space.

Efforts to gauge seed industry performance might seek to collect and monitor several key indicators. First, they might focus on compiling and analyzing data along the lines of DIIVA, TRIVSA, and LSMS-ISA to characterize diffusion and adoption patterns and providing nuance to the generally non-descript, aggregate figures on demand, supply and replacement rates described earlier. Such data can also be used to identify spatial, temporal, and distributional dimensions of diffusion and adoption patterns at a resolution that is otherwise absent in government statistics aggregated by state/province or district levels.

Efforts to gauge seed quality are slightly more challenging, but not prohibitively so. In fact, infrastructure exists in many developing countries to assess seed quality. Public research organizations and seed certification/quality assurance agencies routinely collect data on purity, moisture, and germination of randomly selected seed lots are often routinely collected by. These same organizations and agencies often collect data on genetic purity and varietal integrity as well, although they likely tend to do so on a more ad hoc or occasional basis as part of maintenance breeding or related research activities.

That said, efforts to collect data on the physical and genetic qualities of seed can be improved by slightly augmenting the routines of public research and regulatory agencies, investing in the provision of requisite personnel and equipment, and taking advantage of the declining costs of new diagnostic tools and technologies. With investment in high throughput systems and sample collection strategies, testing procedures could be scaled to levels that provide effective monitoring of seed quality at a national level (e.g., ASTA (American Seed Trade Association), 2011, ISU-STL (Iowa State University Seed Testing Laboratory), 20). And as the costs of advanced diagnostics come down, investments in high throughput genetic fingerprinting systems become equally viable, as demonstrated by Westengen et al. (2014) for maize in Tanzania and Rabbi et al. (2015) for cassava in Ghana. Furthermore, by shifting from quality control systems that rely on monitoring at all key points of the seed production process to a more straightforward system of point-of-sale inspection system, there is scope for significant efficiency gains in quality assurance.

These systems would also help address the non-trivial concern that farmers are unable to identify or misidentify the variety they are cultivating when responding to a household survey modeled along the lines of LSMS-ISA or similar surveys. Thus, efforts to compare and validate farmer-reported variety information with alternative diagnostics such as expert assessment or genetic fingerprinting can help determine the nature and direction of bias (see Rabbi et al., 2015).

By combining variety-level data on marketing, adoption and seed quality with spatial and household data, there is considerable scope to improve the quality of evidence used in decision-making on research priorities, public input provision programs, incentive mechanisms, and market interventions. Indicators such as those described above can be useful in strengthening the evidence underlying narratives formed by various seed system actors. With additional data and analysis on innovation, industry structure, and regulation—topics that are explored in the next sections—seed system actors can potentially inform and inform policymaking more effectively.

### 3.3 Seed industry innovation

In addition to quantity and quality data, precise data on cultivar improvement activities are critical to understanding the rate of innovation for a given crop which, in turn, allows analysts to gauge a seed system's capacity to deliver modern science to farmers and enhance agricultural productivity. Many of the performance indicators mentioned earlier—area planted to improved varieties/hybrids, changes in area planted over time, and estimations of the benefits associated with adoption—are used on an occasional basis to gauge innovation, but face measurement and methodological challenges (Morris and Heisey, 2003, Alston et al., 2011). Other approaches rely less on single indicators and more on industry analysis, for example, in case studies of public and private innovation patterns and trends in Asia's seed markets (e.g., Pray and Fuglie, 2001, Pray and Ramaswami, 2001, Pray and Nagarajan, 2012, Gisselquist et al., 2013, Singh and Pal, 2015). However, regular and systematic data collection efforts needed to augment these approaches remain rare. Public disclosures of key indicators such as the number of varieties/hybrids released, the year of release, and their production quantities (Table 3) are still difficult to access, although some government agencies are making these data available more accessible online. See, for example, the expansive datasets posted to the Seednet India Portal, an initiative designed and developed by the National Informatics Centre (Seednet, 2015).

Apart from SeedNet, TRIVSA (mentioned earlier), occasional publications from CGIAR centers,^6^ and a few similar initiatives, innovation indicators are difficult to come by. One notable exception is the Agricultural Science and Technology Indicators initiative (ASTI, 2014), which collects and analyzes data on public investment in agricultural research using an approach consistent with the Organisation for Economic Co-operation and Development's Frascati Manual (OECD, 2002). However, ASTI coverage is limited to R&D inputs—financial and human resources—such that it does not capture innovation outputs such as new crop- and variety-specific products, processes, and services. Moreover, ASTI indicators concentrate primarily on public sector spending: while ASTI does contain data on private R&D investment for certain countries in certain years, the data are rarely at a level of disaggregation comparable to its data on the public sector. Other sources of private sector data and analysis do exist, for example, McDougal (2015) in the U.K. and Francis Kanoi (2015) in India. However, their proprietary information products are often too costly or otherwise inaccessible to public sector analysts, regulators, or policymakers.

That said, there are readily available and low-cost ways of constructing indicators that provide a sense of seed system innovation. The most common is an index of varietal age for a given crop, which is calculated as the average age of varieties in production weighted by the quantity of production.^7^ A higher average age is associated with a low rate of varietal turnover and, implicitly, a slow rate of innovation in the seed industry (Smale et al., 2008, Brennan and Byerlee, 1991). Historically, these measures have been calculated for wheat and rice using variety-specific seed production data from state-owned seed companies (see, e.g., Lopez-Pereira and Morris, 1994). However, with additional variety-specific data on private sector production, a more complete indicator can be calculated for maize, as demonstrated in Table 3.

Importantly, this measure can also be calculated for the average age of varieties under cultivation at the farm level by using crop/plot- and variety-specific data from representative household surveys. Ragasa et al. (2013) demonstrate this for maize in Ghana, as do Krishna et al. (2015) for wheat in Haryana, India. But there is potential for expanding this analysis further using LSMS-ISA and other agricultural household surveys containing variety-specific questions mentioned earlier, but also other agricultural household surveys that contain variety-specific questions. Information on the average age of varieties under cultivation—especially when provided in a spatially disaggregated manner and correlated with social and economic attributes of the household, farm, and market—can provide useful insight into the heterogeneity in innovation among populations targeted by the seed industry. That said, it is also difficult to obtain accurate variety-specific responses in such surveys because of poorly pre-coded lists of variety names in survey instruments, or poor recall by farmers. This highlights the need for better survey design incorporating local knowledge, and, as mentioned earlier, the potential use of low-cost genetic diagnostics as a validation tool.

### 3.4 Seed industry structure

Indicators of seed industry structure are another potentially important means of understanding the relationship between competition and innovation which, in turn, can inform analyses of the seed system's capacity to deliver modern science to farmers and enhance agricultural productivity. The conventional measure of market structure is often source of seed—estimated quantities and shares of seed that are purchased from formal versus informal sources (disaggregated by crop) and, within the formal sector, seed that are purchased from public sources versus private firms and community, farmer, or civil society organizations. This measure provides a simple indicator of the size of the formal, commercial seed industry and its growth potential.^8^ Of course, this assumes that commercial seed is a viable substitute for farmer-saved seed which, as noted earlier, may not always be the case.

While these indicators are useful in helping us understand the source of seed, they are often too aggregated to provide real insight into market concentration. Instead, consider two common indicators used to measure market concentration: four- and eight-firm concentration ratios (CR4 and CR8) and the Herfindahl–Hirschman Index (HHI). The CR4 and CR8 ratios measure the total market share held by the four or eight largest firms in the industry, respectively. The HHI measures the size of firms in relation to the industry and is calculated as the sum of the squared market share (in percentage terms) of each firm in the industry.^9^ The HHI approaches zero when a market consists of a large number of firms of relatively equal size, and increases both as the number of firms in the market decreases and as the disparity in size between those firms increases. Because the HHI takes into account the relative size and distribution of the firms in a market, it is considered a more comprehensive indicator of concentration than the CR4 and CR8 ratios (Scherer and Ross, 1990, Rhoades, 1995).

These indicators may be used to measure concentration in downstream product markets using the value or volume of seed sales by firms in the seed industry (Fernandez-Cornejo, 2004), or in upstream innovation markets using the number of varieties under development or field trial applications approved (Brennan et al., 2005). While indicators from a single year can be useful to gauge concentration, trend data provide greater analytical insight (e.g., Fuglie et al., 2011, Fernandez-Cornejo, 2004).

An illustration is given in Table 4 for Nepal, indicating moderate concentration in country's maize market, particularly when compared to rice. A similar illustration for India's innovation market where *prospective* technologies are developed for the seed market is provided by Spielman et al. (2014) using data from Randhawa and Chhabra (2009) on field trials and transgenic material imports attributable to the private sector to calculate concentration ratios. Their figures suggest that market concentration in India's agricultural biotechnology sector decreased between 2006 and 2010, a trend that is likely explained by the entry of new firms into the field of biotechnology research. More importantly, these calculations demonstrate the feasibility of measuring and characterizing concentration in both product and innovation markets.

A related indicator is the extent to which government participates in the seed market and how the gains and losses associated with such participation are allocated between seed companies, farmers, and other seed system actors. Interventions include direct engagement in seed production and distribution through state-owned seed enterprises and extension services which, under certain circumstances, can impede private sector entry and participation in the seed market. More indirect interventions include the provision of specific advantages to state-owned enterprises or selected private firms, such as production subsidies, tax breaks, preferential access to improved germplasm from the public research system, well-endowed land for seed production, subsidized credit, credit guarantees, tariff exemptions on equipment imports, and other benefits that lower seed production and distribution costs. Other interventions may take the form of direct subsidy payment to farmers purchasing seed from state-owned enterprises or selected firms.

Many developing countries in Asia have dismantled the large input subsidy regimes that underwrote rapid productivity growth in food staples during the 1960s and 1970s, although some subsidies persist e.g., water and electricity in India or fertilizer in Pakistan (Anderson, 2009, Anderson and Martin, 2009). Seed subsidies are also somewhat resilient to policy changes, possibly because they are a low-cost intervention relative to subsidies for bulky inputs such as fertilizer, or because they are conventionally viewed as a critical means of encouraging farmers to experiment with new varieties. However, there is a notable absence of data on seed subsidies and other interventions in seed systems that distort market signals. Without such data, it is difficult to calculate the price elasticity of demand for seed and answer common questions such as whether seed providers raise their prices in close proportion to the subsidy amount, or whether farmers have bargaining power in these markets. And without such answers, policymakers can rarely make evidence-based decisions on the optimal allocation of public resources for seed industry development.

Yet in many cases, the data described above can be collected with a relative ease from the same sources discussed earlier: public documents on government investments and programs in agriculture, household and market/industry surveys, and expert opinion surveys. But in instances where detail and nuance matter, case-study approaches are likely to be more appropriate. Business school case studies, industry analyses, and other qualitative approaches can provide greater insight into how public and private organizations conduct R&D, how they organize seed distribution and marketing, or how they navigate weather risk, market distortions, and regulatory systems. See, e.g., Rabobank (2006) on India's seed industry.

### 3.5 Seed market regulation

Indicators of the presence and effectiveness of seed industry regulations offer another potentially important means of understanding a seed system's capacity to deliver modern science to farmers and enhance agricultural productivity. Unfortunately, regulations governing seed markets have not been methodically compiled for most developing countries. An early indication of the feasibility of collecting these indicators is demonstrated in a 10-country pilot study conducted by the World Bank Group (2014) that is loosely modeled on the “Ease of Doing Business” report series of the World Bank (2015),^10^ but focuses explicitly on identifying and monitoring indicators that capture data on 19 seed systems regulations and policies that are posited to enable the business of agriculture. Another indication of feasibility is found in GRAIN's (2015) interactive atlas that highlights seed laws around the world, focusing specifically on the nature and extent of farmers' rights in each country. We extend insights from these sources by describing below regulatory indicators covering varietal registration, seed certification, intellectual property rights protection, and biosafety regulation that may be useful in the analysis of developing country seed systems.

#### 3.5.1 Varietal registration and seed certification

Historically, when the majority of field crop varieties were developed by public research systems, regulations were codified in varietal registration procedures that were relatively standard processes for public breeders to navigate. As privately developed varieties—including privately developed maize hybrids—became increasingly available, countries have had to decide how to adjust variety release and registration requirements to accommodate this wider offering. Regulatory responses have ranged from a single testing and release system for all varieties to allowing private varieties to enter the seed market without any release requirements.

To illustrate the importance of regulatory system analysis, consider the policies and procedures found in several of the Asian countries highlighted in this study. While these countries shared a common set of procedures that revolved around value in cultivation and use (VCU) testing and testing for distinctness and uniformity and stability (DUS), there is significant variation in where the procedures are applied and the time involved. For example, whereas extensive VCU and DUS testing are required for all cultivars in most countries, Bangladesh does not require extensive testing for maize: testing is only required for rice, wheat potato, jute, and sugarcane, which represent the strategically important “notified” crops that are required to undergo close government scrutiny. And whereas Bangladesh and Vietnam have relatively short testing periods of just two to three years, countries such as Nepal can require a full five years of testing.

Next, consider seed quality control regulations—an area with similar levels of variation between countries. For example, notified crops in India, Bangladesh, and several other countries, must undergo certification processes that include examination of the documentary evidence regarding the source of the seed for the current seed crop, several inspections of the seed crop in the field, and sometimes grow-outs or laboratory analysis of harvested samples. In many of these same countries, non-notified crops can move to market as quality declared or truthfully labeled seed: regulatory regimes that shift the burden of quality control to the seed provider, whose business success may depend on building brand reputation and securing repeat clients; or the farmer, who can pursue legal recourse for seed that does not meet the advertised characteristics of physical or genetic purity.

Certification—the higher quality control standard—is expressly designed to address the asymmetries of information between seed provider and farmer, i.e., the fact that it is often difficult for farmers to assess the identity or quality of seed upon visual inspection. But this does not necessarily guarantee that certification or similarly stringent regulations actually assure the supply of seed relative to other regulatory options. Companies may choose a higher standard of self-regulation to protect their brand, or the seed industry may self-regulate collectively to exclude companies they consider to be lower quality providers, competitors, or otherwise undesirable. It is also possible for public regulators and public quality control agencies to share duties, for example, by accrediting seed companies or third-party laboratories to carry out a majority of required inspections while allocating public agency with responsibility for reviewing company inspection data, conducting independent point-of-sale inspections, or holding an oversight role. Rana (2014) highlights these issues in Pakistan, where the absence of effective quality control in the cotton seed sector calls into question the very utility of the country's seed certification system. This is in stark contrast to Pakistan's experience with maize, where quality control seems to be driven by a single market leader—Pioneer, a leading multinational company—seeking to protect the reputation of its brand.

Measurable indicators of a country's regulatory environment need to both describe the registration and quality control process and assess its effectiveness. Indicators on registration procedures include answers to these questions: (a) which crops must be registered; (b) how many years of testing in how many locations are required before a variety can be released; (c) If private varieties must go through the same process, are they treated on an equal basis with public varieties; (d) are imported private varieties given equal footing to public or private varieties developed domestically; (e) are public varieties that have been released in other countries with similar ecologies offered a fast-track to release?^11^ Similarly, indicators on quality control procedures include answers to questions such as: (a) which crops fall under certification or truthful labeling regulations; (b) are inspections conducted by a public agency, by companies themselves, or by third parties; (c) are inspections conducted during the production process or at point-of-sale, at what frequency and geographic/market coverage, and with what size inspection force; (d) at what rate are seed lots rejected under these various inspection regimes, and how frequently are inspections; and (e) how many instances of legal recourse against seed providers have been pursued in the courts, how transparent are the procedures, and what levels of sanctions are imposed? Additional questions might focus on trade regulation issues, for example: (A) can breeders easily import genetic materials from foreign sources, and can they share domestic material with foreign breeders; (b) do plant sanitary and phytosanitary regulation and plant quarantine procedures exist, and are they effectively implemented; and (c) are there laws in place to prevent unsanctioned imports such as seed trade across porous borders?

Answers to these questions—garnered from government documents, corporate disclosures, or expert interviews—can provide a set of individual indicators or the basis for calculating a composite index of regulatory coverage and effectiveness. On its own, such indicators or an index can provide a potentially useful means to explore associations between innovation, productivity, and regulation in developing country seed systems. The pilot study by the World Bank Group (2014) demonstrates the feasibility of gathering data for a subset of these indicators; however, the limited variation in their data suggest the need for greater detail.

#### 3.5.2 Intellectual property rights and biosafety

There is an extensive literature predicting that the entry and growth of private R&D investment in developing-country agriculture will hinge significantly on intellectual property rights, particularly in cases involving advanced biotechnology tools and products (Byerlee and Fischer, 2002; Pingali and Traxler, 2002). However, there are also predictions that IPRs, by providing private firms with temporary monopolies, may limit smallholder farmers' access to new technologies in developing countries (Goeschl and Swanson, 2000; Srinivasan and Thirtle, 2000). Others suggest that IPRs are inconsequential to smallholders in developing countries because firms rarely seek IPRs in markets offering relatively limited value or where other means of IPR protection such as hybridization might exist (Binenbaum et al., 2003; Spielman and Ma, 2015). There is some evidence of the impact of IPR protection on innovation and productivity (Kanwar and Evenson, 2003, Kolady et al., 2012). However, others have found the evidence to be mixed at best (Naseem et al., 2010; Spielman and Ma, 2015). Yet despite the ambiguous nature of the evidence, IPRs still loom large in the policy discourse on developing country seed systems. Thus, there is utility in benchmarking the presence and effectiveness of their IPR regulations, even if as a means of later exploring their contested effect on innovation, competition and productivity.

A simple measure of IPR regime strength might be to determine whether a given country has enacted legislation that extends IPRs over plant varieties in a manner that is compliant with the agreement on Trade-Related Aspects of Intellectual Property Rights (TRIPS); whether the country has joined the International Union for the Protection of New Varieties of Plants (UPOV); and whether the country opted for the UPOV 1991 or 1978 Act, where the latter contains language that expressly protects farmers' privilege. Additional measures might reference the presence of patent laws and other IPR legislation designed to provide firms with the right to protect genes, gene sequences, tools and processes used in the development of transgenic crops. More complex measures might capture the extent to which innovators have sought recourse for IPR infringements in the courts, or the extent to which rules and regulations protect farmers' rights to save, exchange, and sell seeds to other farmers, or seek IPR protection over extant varieties (e.g., GRAIN, 2015).

The Ginarte-Park Index of IPR regime strength provides a useful model for what might be calculated with specific reference to developing-country seed systems and agriculture more generally (Ginarte and Park, 1997). The index assesses IPR regime strengthen on a scale of 0 (a weak regime) to 5 (a strong regime) based on indicators of IPR coverage, duration, enforcement, and limitations. In its most recent version, the index provides coverage of 121 countries between 1960 and 2005 in five-year increments (see Park and Wagh, 2002, Park, 2008). Other indices such as Rapp and Rozek (1990) are also viable models.

Several Asian countries have also introduced legislation that protects plant breeders' rights through PVP and/or *sui generis* (standalone) systems, are moving towards compliance with TRIPS, and have signed on to UPOV 1991 as part of their commitment to plant breeders' rights (Table 5). But India's experience may eventually emerge as the most useful case study of whether the measurement of IPR protection matters. The hallmark of India's seed policy regime is the Plant Varieties and Farmers' Rights (PPV&FR) Act of 2001, designed to incentivize private investment in plant breeding while simultaneously protecting farmers' privilege. With the establishment of the PPV&FR Authority in 2005 and the commencement of varietal protection application processing in 2007, both the public and private sectors have submitted applications for protection of outputs from their breeding programs (Table 6). These data provide a clear indication of how innovators have responded as expected to the regulatory regime. Efforts to obtain additional data on whether India's courts have adjudicated on infringement cases and, if so, whether the outcomes of their decisions have had an impact on the private R&D investment, would be a valuable addition. By tying these indicators together and analyzing them in the context of other indicators discussed in previous sections, it is possible to paint a more accurate picture of the influence of IPR regulation on innovation, competition and productivity.

Next, we turn to biosafety regulation. The relevance of biosafety regulation to a given country's seed systems is contingent on broad policy decisions made about whether to cultivate transgenic crops or not. In many countries, that decision is *de jure*, and influenced by the relative strength of competing—and highly contested—narratives around the introduction of genetically modified crops and other organisms. But in other countries, the decision may be influenced by the *de facto* presence of unapproved transgenics.

Whether transgenic commercialization is de jure or *de facto* in a given country, seed industry development requires national capacity to provide an appropriate biosafety system to evaluate the consequences of transgenic crop releases to human and environmental health. Conversely, the absence of such systems can quickly undermine the prospects for transgenic crop improvement, especially when conflicting advocacy coalitions with distinct narratives collide over the design and implementation of biosafety regulations (Kingiri, 2011).

Indicators on the status of biosafety legislation governing the commercialization and release of transgenic crops, as well as the financial and technical capacity to enforce such legislation, can provide useful insights into the enabling environment for innovation in a rapidly growing niche of Asia's seed market. As of 2015, 12 Asian countries had approved genetically modified events for maize (Table 7) (ISAAA, 2015). What is absent, however, is a set of indicators that capture the presence and effectiveness of this biosafety system—indicators that capture uncertainties such as those in India, as well as progress toward the creation of an environment designed to ensure safe and effective use of biotechnology in agriculture. FAO, for example, maintains an inventory of national biotechnology strategies and policies (FAO, 2015). Such inventories could be one of several elements in a biosafety index similar to the Ginarte-Park Index mentioned earlier. Ideally, the index would integrate data on the existence, implementation, and efficacy of public policies and regulations on the safe use of biotechnology applications in agriculture, and would be a relatively low-cost investment in country-level collection of secondary data that is easily updated on a period basis.

## 4 Discussion: where data and analysis might inform policy discourse

The current indicators being used to measure performance, competition, and innovation in the maize seed sector fall short of measuring these critical industry characteristics on many counts. The indicators suggested above provide greater resolution at various levels—spatial, social, household, farm, plot and varietal—and represent a first step towards more methodical analysis of the opportunities and tradeoffs in seed system development. Such analysis can be used to shape national agricultural growth strategies, set public research priorities, design private innovation incentives, construct public input provision programs, and encourage maize seed industry development and productivity-enhancing technology adoption. In this section, we highlight several instances where current policy discourse on seed system development in Asia would benefit from such analysis.

India provides a useful starting point given its considerable experience with input subsidy programs to accelerate technology adoption and productivity growth. Under the National Food Security Mission (GOI, 2007), India's most recent agricultural sector support initiative, farmers purchasing seed for certain types of crops (e.g., hybrid rice) receive direct subsidies, reportedly up to 50% in some states. Yet anecdotal accounts of seed sellers increasing their retail prices in response to the availability of these subsidies suggest that the scheme may be poorly designed and targeted. Estimates of price elasticities of demand and supply extracted from household and market surveys, combined with distributional analyses of adoption patterns extracted from household surveys, could be used to furnish evidence on the effectiveness of such schemes and their inherent tradeoffs.^12^ Such analysis could, in turn, influence the design of better targeting mechanisms and better uses of public funds.

Policy discourse in Bangladesh offers a similar opportunity for greater use of evidence. At present, the state-owned Bangladesh Agricultural Development Corporation (BADC) plays a central role in the distribution and marketing of improved cultivars through an extensive production infrastructure and its large network of shops and authorized retailers (Ar-Rashid et al., 2012, Naher and Spielman, 2014). It also retains a significant portfolio in seed for several crops that might be more appropriately handled by the private sector, including hybrid rice and maize. Given that BADC's operational costs are partly underwritten by scarce public resources, a closer analysis of market concentration and industry structure using the indicators suggested above could help inform discussions about rationalizing BADC's role and shift it into a more strategic position that supports the country's growing private seed sector. This was the original but partly unrealized intent of the 1993 National Seed Policy, provisions of which called for the opening of equitable opportunities to the public and private sectors at all stages of the seed industry.

Similar evidence on market concentration and industry structure could be used to inform some of Asia's more contentious policy debates around the equitable distribution of gains from innovation between farmers and seed companies and the related issues of seed sovereignty, foreign direct investment, and multinational company participation in domestic seed markets. This debate has been central to public discourse around imports and promotion of hybrid maize in Nepal and the role of donor-funded projects involving multinational cropscience companies (see, e.g., Nepali Times, 2011, SciDev.net, 2011). Similar debates on distributional issues and the multinationals are ongoing in Pakistan (GRAIN et al., 2010) and the Philippines (Kuyek, 2000), among others (GRAIN, 2005), but are often absent of evidence. Measurements of industry concentration in innovation and product markets could help monitor changes in market concentration and detect anti-competitive practices.

Evidence on regulatory system effectiveness and analyses of the costs and benefits of regulation could also play an important role in informing policy debates in Asia. In Pakistan, for instance, a policy debate is emerging around the 2015 Amendment to the Seed Act of 1976 and a proposed act on plant breeders' rights. Both pieces of legislation seek to recognize and open the seed market for the private sector, but offer little in terms of regulatory reform. Rather, the amendment to the Seed Act would extend the federal government's regulatory authority and reach over the private sector, without sufficient recognition of the associated costs (and benefits) of a stronger regulatory authority when compared to alternatives such as a truth-in-labeling regime (See Rana et al., forthcoming). A more evidence-based debate could reduce the dependence on conjecture and speculation these situations.

Similarly, stakeholders in debates over genetically modified crops would benefit from a keener sense of precisely how comprehensive their country's biosafety regulations are in addressing human and environmental health risk, and whether the scientific, technical, and administrative capacity exists to implement these regulations. In Nepal, the policy debate on hybrid maize described above would have benefited from this type of information when several narratives emerged that incorrectly conflated demonstration trials of imported hybrid maize with the introduction of genetically modified crops, loss of national sovereignty to a multinational company, and abrogation of Nepal's commitments to international treaties on plant genetic resource use and conservation (Nepali Times, 2011, SciDev.net, 2011). A similar use of evidence could have informed debates in countries that approved the cultivation of selected GM crops (Bt eggplant in Bangladesh; Bt maize in the Philippines and Vietnam), in countries that have pursued biosafety approvals for GM crops but stopped short of commercialization (Bt eggplant in India; Bt rice in China), and in countries that have not yet reached a decision beyond a single crop and a single class of technology (Bt cotton in India and Pakistan).

In India, for example, the policy debate would have benefited from a better and earlier sense of whether the country's biosafety system had sufficient competency and capacity to fulfill its mandate. Answers to this question—whether in the form of qualitative or quantitative evidence—could have shaped narratives and informed decision-making in two instances. This could have occurred as early as 2001, when regulators had to decide how to handle the approval of Bt cotton after unapproved varieties were already detected in farmers' fields; and later, in 2010, when the Minister of Environment issued a moratorium on the commercial release of Bt eggplant, despite the presence of a national biotechnology development strategy, approved biosafety review procedures, accumulated experience with Bt cotton, and official approval for commercial cultivation by the appropriate regulatory committee (Herring, 2007, Kolady and Herring, 2014). A similar experience with Bt cotton in Pakistan in which the technology became available to farmers several years before the biosafety regulatory system issued its approval, similarly suggests that better data and analysis on regulatory efficacy could have been useful in shaping the continuing debates about subsequent Bt cotton approvals in Pakistan (Rana, 2014, Spielman et al., 2015).

Necessarily, indicators and analysis do not alone effect policy change: the implementation of policy reforms and the conduct of regulation are determined by a complex landscape of political economy factors in a given country. As such, this discussion is only meant to demonstrate the potential role that evidence can play in promoting seed industry development and improving the delivery of modern science to farmers and enhancing agricultural productivity. It is meant to help guide decisions on investment and regulation, rather than provide definitive guidelines for measuring, regulating, and governing seed systems.

## 5 Conclusion

This paper explores the measurement of performance, innovation, and competition in developing-country seed systems to better inform policymaking aimed at improving the delivery of productivity-enhancing modern science to farmers. Drawing on experiences from selected Asian countries, the paper demonstrates that while policy reforms introduced beginning in the 1980s have led to seed industry growth in many countries, opportunities for subsequent policy changes is partly constrained by the absence of analyses utilizing indicators that adequately capture the trade-offs associated with policies meant to effectively govern a modern seed industry. With better measures of the seed industry health such as high-resolution data on productivity and distributional aspects of performance, analyses of innovation pipelines, products, and processes, and indicators on concentration and competition, policymakers can more accurately assess available policy options. Necessarily, policymaking does not occur in a vacuum, and policymakers must contend with competing narratives, each with distinct and varying goals, values, and levels of influence. Yet better data and analysis are also central to formulating and communicating these same narratives.

Several recommendations do emerge from this discussion. First, there is clearly scope for expanding the systematic collection and analysis of data that are relevant to seed systems in developing countries. Initiatives such as DIIVA, TRIVSA, and LSMS-ISA, are all examples of data-intensive efforts that contribute along these lines and could be expanded. Second, there are opportunities to use new tools in genetic diagnostics to develop new quality indicators or validate existing indicators to improve seed system performance measurement. Third, there is scope to build regulatory indicators and indices covering individual components of a country's seed regulatory system—for example, registration, quality control, IPRs, and biosafety—or the system as a whole. The Ginarte-Park Index offers a useful model, as does the pilot study by the World Bank Group (2015). Finally, there are opportunities to make these data available and accessible to researchers and decisionmakers in the public, private and civil society sectors to better inform policymaking.

## Figures and Tables

**Table 1:** Estimated seed demand and supply from various sources for selected Asian countries, metric tons (mt).

Country (year)	Crop	Estimated total seed demand (mt)	Production (mt)			Production as a share of estimated total seed demand (%)		
Public	Private	Informal^a^	Public	Private	Informal^a^
Bangladesh	Maize	5000	288	4512	200	5.8	90.2	4.0
(2012)	Rice	319,500	181,428	6392	131,680	56.8	2.0	41.2
	Wheat	55,700	39,840	0	15,860	71.5	0.0	28.5
Pakistan	Maize	31,914	245	3460	28,209	0.8	10.8	88.4
(2012)	Rice	42,480	5068	40,699	3610	11.9	95.8	8.5
	Wheat	1,085,400	72,112	187,792	552,180	6.6	17.3	50.9
Thailand	Maize	23,945	955	22,990	0	4.0	96.0	0.0
(2012)	Rice	1,009,230	245,000	300,000	455,000	24.3	29.7	45.1
Vietnam	Maize	21,358	19,200		2158	na	na	10.1
(2012)	Rice^b^	882,750	233,850		648,900	na	na	75.0

Sources: Bangladesh: Naher and Spielman (2014); Pakistan: Rana (2014); Thailand: Napasintuwong (2014); Vietnam: Nguyen Mau Dung (2014).

Notes: ^a^ “Informal” denotes farmer-saved seed and seed purchased through informal markets and farmer-to-farmer exchanges. ^b^ Includes both inbred and hybrid rice.

**Table 2:** Indicators of seed industry performance, innovation, structure and regulation.

Indicator domain	Suggested indicator (unit)	Level of disaggregation
Industry performance	Seed sales (metric ton)	Geo-referenced at lowest level possible
	Seed prices (local currency)	Geo-referenced at lowest level possible
	Seed quality -Germination, moisture, purity -Genetic and trait purity	Variety-specific samples of individual seed lots
Innovation	R&D spending	By sector (public/private)
	Varietal releases	By producer/source
	Age of varieties in production	By producer/source
	Age of varieties under cultivation	Spatial, social, and economic disaggregation using household data
Structure	Seed sources -Formal vs. informal markets -Public vs. private providers	By crop
	Innovation market concentration -HHI, CR4, CR8 measures	By crop
	Product market concentration -HHI, CR4, CR8 measures	By crop
	Distribution network structure	By crop
	Market distortions -Market share of SOEs -Producer, consumer subsidies -Tax credits, export subsidies, tariffs	By crop
Registration and quality control regulations	Variety release requirements -Procedures, duration, exemptions	By crop
	Seed quality and certification -Procedures, duration, exemptions	By crop
	Seed inspection procedures	By producer, market or region
Intellectual property rights and biosafety regulations	Plant variety protection applications	National
Patent applications	National
Compliance with TRIPS	National
Membership in UPOV	National
Existence of biosafety regulations	National
Implementation capacity/expertise	National

Source: Authors.

Notes: HHI denotes Herfindahl–Hirschman Index; CR4/8 denotes four and eight-firm concentration ratio, respectively; SOEs denotes state-owned enterprises; TRIPS denotes the agreement on Trade-Related Aspects of Intellectual Property Rights; UPOV denotes the International Union for the Protection of New Varieties of Plants.

**Table 3:** Varietal releases for selected crops, years and countries.

Country	Crop	Years	No. of varieties released		Average no. of releases per year	
Public	Private	Public	Private
Bangladesh	Maize (all)	1994–2011	19	98	1.0	5.2
Indonesia	Maize (composite)	2006–2012	8		1.1	
	Maize (hybrid)	2006–2012	82		11.7	
Pakistan	Maize (all)	1990–2013	16	2	0.7	0.1
Vietnam^a^	Maize (all)	1977–2012	118		3.3	

Source: Bangladesh: Naher and Spielman (2014); Indonesia: Jamal (2014); Pakistan: Rana (2014); Vietnam: Mau Dung (2014).

Notes: ^a^ Figures for Vietnam are only available as combined totals of all (public and private) releases.

**Table 4:** Concentration in Nepal's seed market, by crop, 2012.

Indicator	Rice	Wheat	Maize
Herfindahl–Hirschman index (HHI)	1294	2185	2070
Four-firm concentration (CR4) ratio (%)	63.6	82.2	90.9
Eight-firm concentration (CR8) ratio (%)	86.9	93.0	99.2

Source: Authors, based on data for Nepal from Sah (2014).

**Table 5:** UPOV membership, selected countries, 2015.

Countries that are members of UPOV (year of joining)	Countries that have initiated the procedure for acceding to the UPOV convention	Countries that have been in contact with UPOV for assistance in the development of laws
Kyrgyzstan (2000)	India	Cambodia
Vietnam (2006)	Tajikistan	Myanmar
	Philippines	Pakistan
		Thailand

Source: UPOV (2015).

**Table 6:** Applications for plant varietal protection, India, 2007–2014.

Crop	Public	Private	Farmer
Maize	117	252	78
Cotton	114	934	1
Rice	242	260	3060
Pearl millet	59	180	3
Sorghum	107	85	29
Wheat	123	18	24

Source: PPV&FR (2014).

**Table 7:** Genetically modified crop events approved in Asia, 2013.

Country	Maize	Other major crops
Bangladesh	No	Eggplant
India	No	Cotton, soybean
Iran	No	Rice
Myanmar	No	Cotton
Pakistan	No	Cotton
Australia	Yes	Alfalfa, canola, cotton, potato, rice, soybean, wheat
China	Yes	Canola, cotton, maize, rice, soybean
Indonesia	Yes	Soybean, sugarcane
Japan	Yes	Alfalfa, canola, cotton, potato, soybean
Malaysia	Yes	Soybean
New Zealand	Yes	Alfalfa, canola, cotton, potato, rice, soybean, wheat
Philippines	Yes	Alfalfa, canola, cotton, potato, rice, soybean
Singapore	Yes	Alfalfa, canola, cotton, maize, soybean
South Korea	Yes	Alfalfa, canola, cotton, potato, soybean
Thailand	Yes	Soybean
Turkey	Yes	Soybean
Vietnam	Yes	None

Source: ISAAA (2015).

## Footnotes

1 These figures are based on assumptions from Fuglie et al. (2011) that maize represents 25% of the global market value for private sector seed combined with more recent figures on the value of the global seed market from Bonny (2014). These figures are greater than R&D spending on other commercial crops such as soybean, cotton, or wheat, and greater than public R&D spending on maize. Among the “big six” multinational cropscience companies (Monsanto, DuPont/Pioneer, Syngenta, Bayer, Dow, and BASF), only two do not invest in maize R&D.

2 For an analysis of how “success stories” come into being in the field of agricultural development, see Sumberg et al. (2012).

3 Gerpacio (2003) makes the important point that as of 1997/98, public investment was still central to maize R&D in many other Asian countries. Among organizations engaged in maize R&D surveyed in her study, 71% were public sector entities, the majority of which were accounted for by China and India. She further finds that the public and private sectors were marketing an almost equal number of maize seed products during the late 1990s, albeit in different backgrounds (open-pollinated and hybrid, respectively).

4 While Flaherty et al. (2013) report that public agricultural R&D spending in the Asia-Pacific region increased by 50% from $8.2 billion in 1996 to $12.3 billion in 2008 primarily driven by the region's low- and middle-income countries, Stad and Rahija (2012), among many others, point out that the region's public research systems face significant challenges with respect to governance, management, and organization.

5 These caveats also apply to open-pollinated maize varieties, particularly those cultivated in Mexico, maize's genetic center of origin and still a source of extensive genetic diversity. See Pixley and Banziger (2002) and Bellon et al. (2006), among others.

6 For example, see the occasional publications on world maize facts and trends from the International Maize and Wheat Improvement Center (CIMMYT).

7 The varietal age index from Brennan and Byerlee (1991) using a weighted average is: ${\mathrm{WA}}_{t}=\sum_{i}P_{\mathrm{it}}R_{\mathrm{it}}$ where *P*_*it*_is the proportion of the area sown to variety i in year t; and *R*_*it*_is the number of years (at time t) since the release of variety i.

8 In some cases, a further level of disaggregation or analytical nuance is required, for instance, in the case of Pakistan where formal seed sources—registered seed companies—informally sell large quantities of unapproved genetically modified cotton varieties through their existing marketing channels (Rana, 2014). This particular case clouds the line between formal and informal seed sources by highlighting a practice in which formal sources are responsible for supplying “informal” (i.e., unapproved transgenic) seed. While it is a rare practice, these issues are not uncommon in other countries where unapproved transgenic cultivars have entered markets through retail operations of known seed companies.

9 Formally, the Herfindahl–Hirschman Index is given as $\mathrm{HHI}=\sum_{i}^{N}S_{i}^{2}\;\;$where *S*_*i*_ denotes the market share of the *i*^*th*^ firm of *N* firms operating in the market. Index values between 1500 and 2500 indicate moderate concentration, and index values greater than 2500 indicate high concentration. The maximum index value is 10,000 and denotes one firm holding 100% of the market.

10 For a critique of this report series, see the Independent Panel Review of the Doing Business Report (2013).

11 For example, Bangladesh, India and Nepal recently signed an agreement that harmonizes rice varietal registration procedures between the countries, paving the way for lower regulatory burdens and more rapid release of improved varieties developed in any of the three countries (IRRI, 2014).

12 A study Rickert-Gilbert et al. (2011) illustrates these trade-offs with Malawi's large-scale seed and fertilizer subsidy program, and is readily replicable in the Indian context.
